# Reflexive eye saccadic parameters in Parkinson’s disease

**DOI:** 10.3389/fmedt.2024.1477502

**Published:** 2024-11-25

**Authors:** Nemuel D. Pah, Quoc C. Ngo, Nicole McConnell, Barbara Polus, Peter Kempster, Arup Bhattacharya, Sanjay Raghav, Dinesh K. Kumar

**Affiliations:** ^1^ School of Engineering, RMIT University, Melbourne, VIC, Australia; ^2^ Electrical Engineering, Universitas Surabaya, Surabaya, Indonesia; ^3^ Goulburn Valley Health, Shepperton, VIC, Australia; ^4^ Department of Neurology, Monash Health, Clayton, VIC, Australia; ^5^ Department of Medicine, Sub-Faculty of Clinical and Molecular Medicine, Monash University, Clayton, VIC, Australia

**Keywords:** Parkinson’s disease, saccadic, latency, eye gaze, reflexive saccade

## Abstract

**Introduction:**

Abnormal reactive saccade, with reduced saccadic gain, impaired smooth pursuit, and unwarranted reactions are clinically used to assess people with Parkinson’s disease (PwPD). However, there are inconsistent findings related to other saccade parameters such as latency and transition times. This study aimed to identify differences in the reflexive saccade parameters of early stage PwPD and aged-matched control (AMC).

**Methods:**

In this observational study, the reactive eye-gaze was recorded for 70 participants (42 PwPD) and parameters of reflexive saccades and eye-gaze fluctuations were extracted. These parameters were then statistically analyzed using the Mann–Whitney U-test.

**Results:**

Results showed that PwPD had significantly shorter latency than AMC for reflexive saccadic movement away from the center of the screen. The overshoot as a fraction of the screen width, a measure of the inaccuracy in reaching the target, was also significantly higher for PwPD. PwPD had greater horizontal and vertical eye gaze fluctuation with a steady target. The numbers of invalid saccades, i.e., when the gaze goes in the opposite direction from the target movement or is considered anticipatory, were similar for both groups; PwPD with 33.43% and AMC with 25.71%.

**Discussion:**

This study shows that there are significant differences in the reflexive saccade of PwPD and AMC measured using an inexpensive eye-tracking device. The presence of invalid saccade trials, and differences between towards and away from screen center, both of which were not considered in earlier studies, may explain the discrepancies between earlier studies. The outcome of this study has the potential to be made a device that would assist neurologists in the assessment of PwPD.

## 1 Introduction

Saccades are fast eye movements to a target position that bring objects of interest to the central visual focus (1, 2) and orient the gaze towards an object of interest. These may be horizontal, vertical, or oblique. However, most saccades are horizontal or near-horizontal as reported by van der Linde et al. (3). They can be voluntary, when scanning a page of text, reflexive to a new visual stimulus, or involuntary during the fast phase of nystagmus.

Parkinson’s disease (PD) is a neurodegenerative disease that affects about 1% of the population above the age of 60 (4). Several oculomotor alterations have been associated with PD. Saccadic hypometria has been found in PD as shown by Pretegiani et al. (5). Many researchers have observed eye saccades features as a potential indication to both diagnose PD and evaluate its progression and treatment effects (1, 6–11).

Basic saccadic circuit involves the cortical and subcortical areas of the brain. The superior colliculus (SC), located in the midbrain, plays a central role in saccadic movements and eye fixation (1, 2, 12–16). The SC receives inputs from the retina, as well as the basal ganglia (BG) and various cerebral cortical regions. This initiates saccadic movements with bursts of neuronal activity that are transmitted to oculomotor control areas. Upon reaching the target, neural integrators generate a tonic burst of activity to keep the eye in a position (2, 17, 18). The burst neurons are then inhibited by omnipause neurons to end the saccadic movement. Although their saccadic neural circuits are similar, the oculomotor control pathways for horizontal and vertical saccades are separate; paramedian pontine reticular formation (PPRF) is recruited for horizontal movements, and the midbrain tegmentum for vertical (2, 19–21). Saccadic initiation is triggered by visual inputs from the optic nerves and modified by motor planning and cognitive inputs from the basal ganglia (BG) and frontal cortical areas. The frontal cortex and cerebellum also have direct influences on pontine burst cells. The dopaminergic deficiency associated with PD would be expected to affect BG control of the saccadic neural circuitry.

Saccadic function can be tested at several levels (1, 9). Reflexive saccades primarily assess motor function, where the participants follow their gaze to a visual target. The other option is an anti-saccadic task (9), which is a voluntary saccade. Instructions are given to move in the opposite direction from the visual target, thus evaluating motor and cognitive functions together with the capacity for inhibitory control. Another is the memory-guided saccadic tasks, which assess spatial working memory, and require rapid eye movements to previously remembered target locations (22). Predictive saccadic tasks, where the subject is expected to move their eyes to a predicted target, assess higher cognitive functions.

Many researchers have reported abnormality in PwPD saccade parameters. Lueck et al. (23) observed abnormality in PwPD saccade movements during memory-guided saccadic tasks, while no impairments were observed in anti-saccadic tasks. Briand et al. (24) did not find any impairment in the reflexive task but PwPD were found to have more latency and made a large percentage of errors compared to the healthy subjects in a voluntary saccade task. Blekher et al. (25) investigated moderate to advanced PD patients and found that voluntary saccades were more impaired in advanced PD as compared to moderate PD. Chan et al. (26) found that PwPD made more directional errors in anti-saccade and memory-guided tasks in comparison to healthy subjects. However, a study conducted by Amador (27) did not show significant differences between PwPD and AMC in anti-saccade tasks. Instead, they reported evidence of execution deficits in PwPD during voluntary and inhibiting reflexive saccades. Van Stockum (28) observed reduced saccade latencies in PwPD during reflexive tasks with the discriminator. Briand (6) detected abnormalities in visually guided and voluntary saccadic movement of PwPD. Waldthaler (29), who observed the parameters of horizontal and vertical reflexive saccades and anti-saccades in 40 patients found that medication did not alter saccade amplitudes and had opposing effects on the initiation of the visually guided saccade (reflexive saccade) and anti-saccade. Contrary to the other researchers, they found that medication such as levodopa increased the reflexive saccade latency.

From the above-mentioned studies, it is observed that PwPD have consistently been found to be more impaired when performing saccadic tasks that involved cognitive functions such as anti-saccades, memory-guided saccades and predictive saccades. However, there is inconsistency in the results for reflexive saccadic tasks, which assess the motoric exhibitory and inhibitory mechanism of BG (1). There may be many reasons under-pinning this such as differences in experimental protocols, equipment used, and participant factors. Thus further experimental investigation is neccessary to better understand the difference between AMC and PwPD on motoric function observed in reflexive saccadic tasks.

This paper reports an experimental investigation of the association of the reflexive eye saccadic parameters with PwPD. The eye gaze data was recorded for PwPD and AMC, and the test was repeated 20 times to determine the repeatability. The parameters were statistically analyzed to identify those with statistically significant differences between the two groups.

## 2 Methods

### 2.1 Participants

This is a case-control observational study in which the eye saccadic data were recorded from PwPD and AMC participants. The inclusion and exclusion criteria for the PwPD were: age between 30 and 85 years, no organic brain syndrome, no pyramidal and cerebellar signs, or uncorrected eyesight. The AMC participants were those between 30 to 80 years of age with no movement or neurological conditions, nor any uncorrected vision problems. All symptoms of AMC were self declared while for PwPD were from the clinical observations by the neurologists.

There were 42 PwPD participants (26 male) and 28 AMC (5 male) who fulfilled the inclusion criterion and volunteered for this study. The PwPD were 70.5±10.4 years old and AMC were 69.7±7.6 years old, with no significant difference(t-test p=0.716). Majority of the PwPD (33 patients) were in their early stage of the disease (disease duration of 5 years or less) (30), and balance in their moderate stage. All PwPD were on their medication and had been diagnosed with PD for 3.9±3.5 years. PwPD were recruited from the Goulburn Valley Health (GVH) Hospital, Shepparton, VIC, Australia, and the Dandenong Neurology & Specialist (DNS) Clinics, Dandenong, VIC, Australia. The GVH hospital is a public hospital and the DNS is a private neurology clinic managed by a team of neurology specialists.

The study and the data collection protocol were in compliance with the Helsinki Declaration of Human Experiments. The data collection protocol was approved by the Human Research Ethics Committee (HREC) of RMIT University (2021-24384-14138 and 2020-19347-11498) and the Goulburn Valley Health (HREC/74760/GVH-2021-258233). Before each data collection experiment, the procedures were explained to the participants and they were required to sign the informed consent.

### 2.2 Device selection

The aim of the project was to develop an affordable method in which clinicians can screen PwPD based on reflexive eye saccade. A set of pilot studies were done and the GP3 Eye Tracker (Gazepoint, Canada) was identified as a suitable device for the purpose because of ease of use, repeatability, and price, which is important for it to be suitable for clinical use.

The eye tracker was placed at the bottom of the laptop screen, with a screen width of 28.5 cm, at 65 cm from the participants’ eye which corresponds to 24.7 degrees of visual angle horizontally. This device has a low sampling rate of 60 Hz which limited the features that could be obtained. More specifically, the tracker could only capture a maximum peak velocity of 300 degree/s while the gaze went from the center to the side of the screen, which is insufficient for the peak saccadic velocity (31). For this reason, the saccadic transition features were not considered in this work.

### 2.3 Eye gaze recording

During the recording session, the participants were sitting comfortably on a fixed study chair in front of a laptop screen located on a study table, while keeping their heads as steady as possible. The GP3 Eye Tracker was placed at the bottom of the laptop screen, 65 cm from the participant’s eye. Before the eye gaze recording, the GP3 Eye Tracker was calibrated using a 5-point calibration method (four corners and the center point). Our pilot study showed that the device was not sensitive to small head movements of the participant.

**Figure 1:**
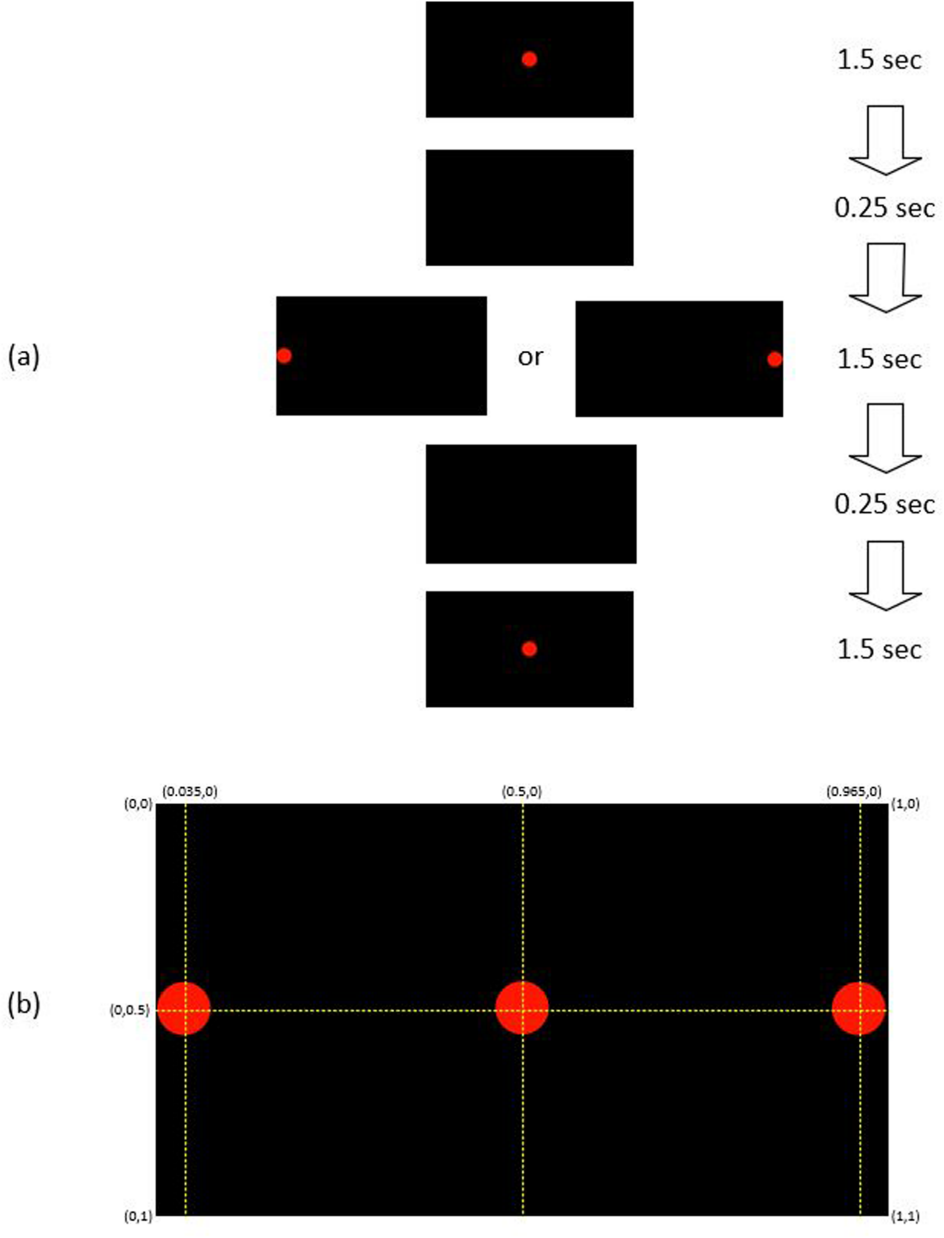
**(a)** The sequence of red dot position: each trial started with a red dot appears at the center for 1.5 s, followed by 1.5 s at the left/right side, and 1.5 s at the center with 0.25 s of blank screen between the changes. **(b)** The coordinate of the red dot position at the center and the left/right side of the black screen.

The participants used their prescription glasses if they needed. They were instructed to focus their eyes on the center of a dot. The diameter of the dot was large (Ø = 2.0 cm or 0.07 of the screen width) such that people with poor eyesight would have no difficulty with the experiment. The color of the dot was red on a black screen, to reduce the background light. The participants were asked to follow the dot when it appeared at the center or the left/right end of the screen as shown in Figure 1. The GP3 Eye Tracker recorded the coordinates of the fixation point of gaze, X and Y (FPOGX and FPOGY), relative to the scale of the screen height and width as in the figure. The validity of the eye gaze is recorded by FPOGV, a Boolean variable; 1 indicates a valid eye gaze. The FPOGV gave a value of zero (invalid) if the eye tracker could not track the eye for any reason or if the participant blinked their eye, and these segments were automatically removed before further analysis. FPOGX and FPOGY coordinates at the instance when FPOGV = 0 were omitted from the analysis.

To test for repeatability, the eye gaze experiment was repeated 20 times, which was based on pilot experiments, where it was found that this was the limit of comfort for most people. Each trial consisted of a red dot position sequence of 1.5 s at the center of the screen, followed by 1.5 s at the left/right side, and then 1.5 s at the center with 0.25 s of blank screen between the changes (Figure 1). There were 20 repeats, 10 to the left (L) side of the screen and 10 to the right (R) side with the sequence of R-R-L-R-L-L-L-R-R-L-R-L-L-R-R-L-R-L-L-R.

### 2.4 Data analysis

Recordings where the gaze was considered anticipatory (with a response time of less than 80 ms), or did not go in the direction of the movement of the dot were classified as invalid. These were noted separately and not used for further analysis. Four saccadic movement features were extracted from the FPOGX and FPOGY recordings. The features were derived into five sub-features as shown in Table 1. The saccadic reaction time (SRT) measures the latency that reflects the ability of the participant to initiate the action in response to a red dot change. The onset of eye saccade was determined as the time the eye gaze left the center coordinate of the screen with a tolerance of 7% (equivalent to the radius of the red dot).

**Table 1:** Description of the extracted features.

Features	Descriptions and Sub-features
SRT	Saccadic Reaction Time: the time elapsed between the appearance of the target and the first onset of eye saccade towards the correct direction (in s).
	∙ SRT_*C − L/R*_ is the SRT when the target is moving from the left to the left/right side.
	∙ SRT_*L/R − C*_ is the SRT when the target is moving from the left/right side to the left.
Sdev	Saccadic Deviation: The distance between the target position and the saccade steady-state position (in proportion to the screen width).
Mean(dx)	Mean of the eye gaze fluctuation in the horizontal orientation while the target is fixed.
Mean(dy)	Mean of the eye gaze fluctuation in the vertical orientation while the target is fixed.

**Figure 2:**
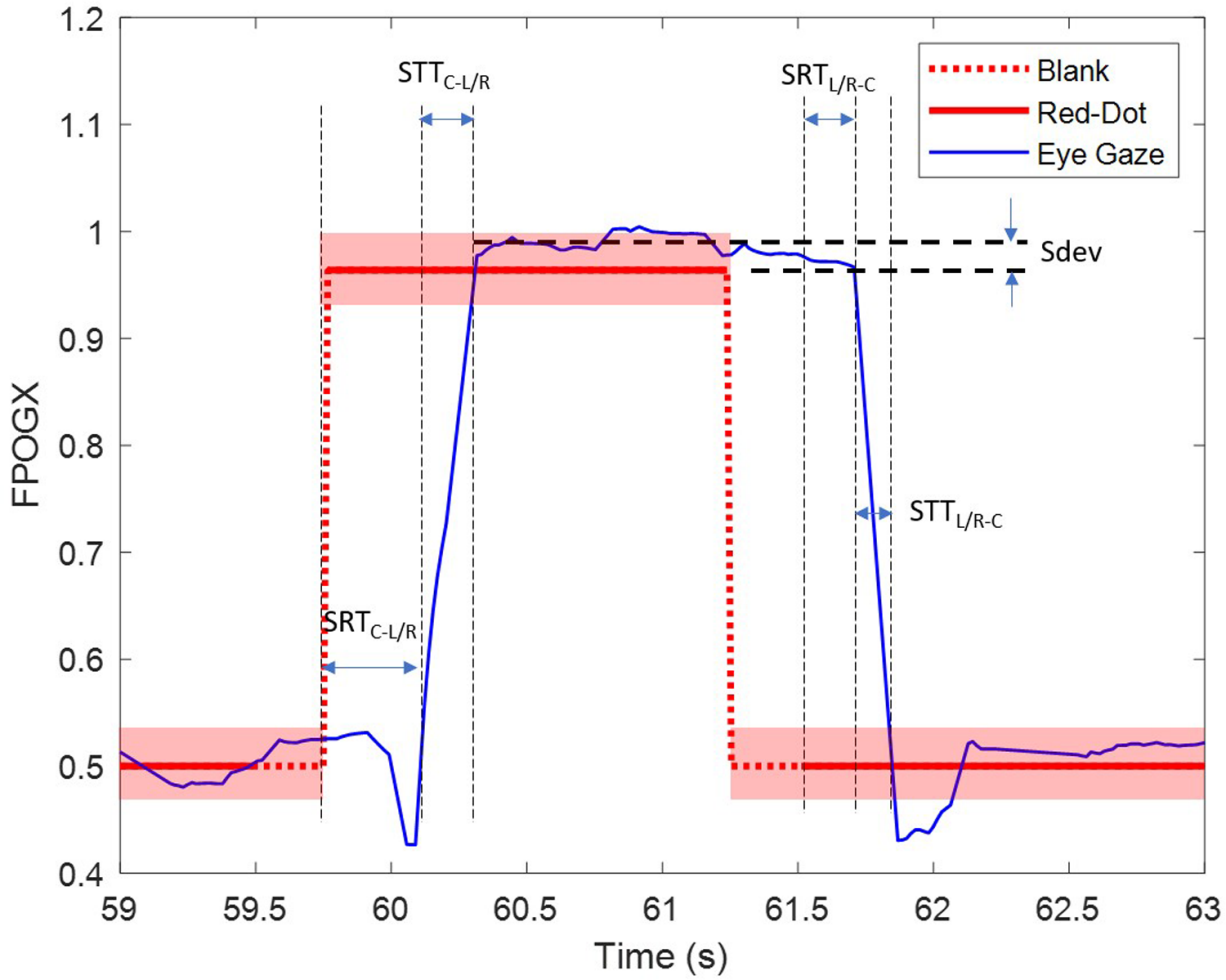
The extracted parameters SRT_*C − L/R*_, SRT_*L/R − C*_ and Sdev for the right trial (R). The transparent red block represent the diameter of the red dot.

The saccadic deviation (Sdev) measures the ability of the participant to accurately direct their eye gaze towards the target. The deviation was only measured in the horizontal orientation (FPOGX). The positive Sdev indicates that the FPGOX was further out to the sides of the screen and vice versa. Figure 2 illustrates the extracted features.

The horizontal and vertical eye-gaze fluctuations measure the ability of the subject to maintain the eye in a fixed position. The features were measured with dx and dy as in the following equations. The features calculated the difference between two consecutive eye gaze coordinates while the red dot target was in a steady position.

(1) $$dx=\{\text{FPOGX}(t_{n+1})-\text{FPOGX}(t_{n})\}{|}_{\text{target steady}}$$

(2) $$dy=\{\text{FPOGY}(t_{n+1})-\text{FPOGY}(t_{n})\}{|}_{\text{target steady}}$$

### 2.5 Statistical analysis

The characteristic of reflexive saccadic features in relation to PD was assessed using statistical analysis. The statistical distribution (mean and standard deviation) of each parameter described in Table 1 was calculated for the two groups, PwPD and AMC. Anderson-Darling test (32) confirmed that the features were not normally distributed, therefore, the Mann–Whitney U-test (33) was performed to compare the differences between PwPD and AMC for the saccadic parameters. Mann–Whitney U-test is a nonparametric statistical test of the null hypothesis that is suitable for data that is not normally distributed. The Mann–Whitney U-test compared the group differences with a 95% confidence level. A p-value of less than 0.05 indicated that the mean of the group was significantly different. The feature extraction and the statistical analysis were performed using MATLAB 2022 (MathWorks, USA).

## 3 Results

### 3.1 Rate of valid saccade

An eye saccade was considered valid if the subject directed their gaze accurately towards the intended direction (left or right), guided by the red dot, and the movement was not initiated while the visual guide was still at its initial coordinates. Overall, 416 saccade trials (74.29%) of AMC and 559 saccade trials (66.55%) of PwPD were valid saccadic movements for the dot going from the center to the sides. For the saccadic movement from left/right to the center, 425 saccade trials (75.89%) of AMC and 544 saccadic trials (64.76%) of PwPD were valid saccades. It was also observed that the first and the last (20th) trials had a number of unsuccessful tests. This could be due to participant fatigue or initial adjustment to the task. Hence, the analysis was performed on trials number 2 to 19.

### 3.2 Saccadic features

Table 2 shows the mean and standard deviation of the saccadic features and the p-value of the statistical Mann Whitney U-test for the 18 trials (trials 2 to trials 19). The reaction time for both groups was higher when going from side to the center compared to center to the side (SRT_*L/R − C*_ > SRT_*C − L/R*_), PwPD SRT_*C − L/R*_ was less than AMC while there was no significant difference between SRT_*L/R − C*_ of PwPD and AMC. This indicates the reduced saccade latencies in PwPD when the target moves away from the center, and no significant difference when going towards the center. The eye gaze reaction of both groups was faster away from the center compared with towards the center. The |Sdev|, which indicates the inaccuracy of reaching the target, was 0.062 for PwPD compared with 0.031 for AMC. This shows reduced PwPD ability to accurately follow the target.

**Table 2:** Statistical distribution of saccadic features in mean(SD).

Saccadic features	PwPD	AMC	p-value
SRT_*C − L/R*_ (s)	0.294(0.044)	0.316(0.044)	0.049
SRT_*L/R − C*_ (s)	0.375(0.069)	0.351(0.066)	0.323
Abs(Sdev)	0.062(0.073)	0.031(0.018)	0.010

### 3.3 Eye gaze fluctuation

The mean of the x and y fluctuation of the eye gazes, i.e., mean dx and mean dy, and the p-value to indicate the difference between PwPD and AMC are shown in Table 3. It is seen that PwPD had higher fluctuation when the visual target was steady, indicating that PwPD were unable to keep steady eye-gaze.

**Table 3:** Statistical distribution of fluctuation features in Mean(SD).

Features	PwPD	AMC	p-value
Mean(dx)	3.625e−03(1.670e−03)	2.818e−03(7.210e−04)	0.033
Mean(dy)	5.276e-03(4.143e−03)	3.802e−03(1.927e−03)	0.067

## 4 Discussion

There are four major findings in this study related to the difference in the saccadic parameters between PwPD and AMC: (1) The saccadic response time of PwPD was faster than that of AMC in the case of reflexive saccadic movements when the target was traveling away from the center. (2) PwPD had higher inaccuracy in reaching the target. (3) PwPD had greater fluctuations with a steady target, and (4) the saccadic features were effective in differentiating PwPD and AMC.

Our study supports the findings of Chan et al. (26), who found that the latency among PwPD was less than those of AMC. They suggested that this was related to the deficit in the PwPD’s ability to inhibit automatic saccades (reflexive saccades initiated by the sudden appearance of a visual target). Briand et al. (6), after comparing the cued vs. uncued response time of PwPD vs. AMC, found that PwPD was faster when cued, but slower when uncued. They suggested that automatic spatial attention processes were more active in PwPD. The performance of PwPD on tasks involving reflexive spatial attention indicated the hyper-reflexivity in PwPD.

However, this is not universally accepted. There are inconsistencies in the results reported in the studies of PD patients’ eye movement on reflexive tasks (1, 11, 29). Some of the studies reported prolonged latency in PwPD when performing visually guided saccadic movements (5, 11, 34–37). This latency was linked to the loss of dopamine neurons resulting in the excessive inhibition of SC neurons by the BG, specifically the substantia nigra pars reticulata (SNr) (37, 38). By detecting and removing the invalid saccades has shown significant differences between AMC and PwPD, which may explain the inconsistencies in previous studies. A systematic literature review needs to be conducted, which will be useful in confirming the source of the inconsistencies.

One shortcoming in earlier research in this field was the small sample size. Our work with 70 participants (42 with PwPD and 28 AMC), is a significantly larger study than most of the similar investigations. We also removed those samples with invalid saccades and thus reduced the variability in the results. Our results support the findings of Chan (26), Brian (6), and Fooken (8), all of whom observed that PwPD participants exhibited more rapid express saccades in the pro-saccade task. Our PwPD were mostly in their early stage of the disease (PD duration of 5 years or less) (30) and this supports the argument made by Pretegiani (5) that reflexive saccades are typically preserved in the initial stages of PD. The latency of reflexive saccades can be maintained during the early stages of the disease, enabling PwPD patients to produce saccades even more rapidly than AMC individuals, particularly for small target eccentricities (5, 37).

The shorter latency in PwPD has also been mediated by higher-order cognitive processes such as attention (39–41). The LATER model (42–45) of saccadic eye movements relates saccadic latencies to the neural threshold to make the decision to move the eye, which is influenced by the level of attention. In our study, we observed that both groups had about 20% to 35% invalid saccades. While this result is consistent with the previous findings (5, 11, 37), however, in this study, the invalid saccade recordings were removed from the analysis to improve the quality of the data. Hence, we are unable to comment on this factor.

The accuracy of the saccadic movement of an individual is the ability to accurately reach the coordinate of the target. Some studies reported hypometric saccades of PD patients (5, 8, 26, 28, 46–48) when eye gaze did not reach or overshot the target position. The phenomenon may be related to excessive superior colliculus inhibition (37). Hypermetric cases in PwPD have been reported by Ba et al. (49), who linked this to the impairments in the inhibition of the reflexive saccade mechanism (37). Our study found that PwPD had a higher saccadic deviation (Sdev) compared to AMC.

Our results showed that there were significantly higher horizontal and vertical fluctuations in PwPD. This could explain why PwPD appears to lack the ability to focus on and scan a scene. It suggests that the tonic burst from the neural integrator (2), which is responsible for maintaining the eye in a fixed position, is impaired in PwPD.

This paper has shown that there are significant differences between the saccades of healthy people and those with Parkinson’s disease. However, it suffers from number of limitations which need to be addressed before its suitability can be confirmed. We have identified four major limitations, and list these below.

The first limitation is that while the selection of the 60Hz sampling was to make this suitable for easy access to clinicians, this has limited the choice of signal features and with a higher potential of error. The second limitation in this study is a gender imbalance in the data; the number of female participants for both, the PwPD and AMC groups is higher than male. While this was despite gender-unbiased participant recruitment, and the study by Wilson et al. (31) suggested there was no significant difference between males and females on any saccade parameters up to 35 degrees, nevertheless this needs to be addressed in future studies. The third shortcoming of this study is that it has only studied PwPD and age matched healthy control, but has not considered people with other neurological or ophthalmological disorders such as Atypical Parkinsonian or optic neuropathy such as glaucoma. These diseases could also alter the saccades which would make the results less specific. Another limitation is that while most of the PwPD were clinically identified to be in the early stage of the disease, the complete UPDRS-III score for some of them was not available. Further, the medicine details were also not available. Thus, the correlation between the severity of the disease, or influence of medication to the saccade parameters could not be investigated. Another limitation is that this is an observational case-control study; a longitudinal study to identify the changes to these parameters with the progression of the disease would be useful. Further, this study has only investigated the reflexive saccade. For a more complete understanding of the problem, voluntary tasks such as the antisaccadic, memory saccadic, and predictive saccades also need to be studied.

This work has the potential for being used to assist the neurologists perform diagnosis on people for detecting diagnosis. However, this needs to be tested for generalisability and for its suitability in the clinical work-flow to ensure it meets the clinical needs.

## 5 Conclusion

This study has determined that PwPD have faster reflexive saccadic response time compared to AMC. They also exhibited higher hypermetric saccades and horizontal and vertical fluctuations when focusing on a steady target. The study has also found that there were a large number (nearly 25%) of invalid saccades, i.e., gaze going in the wrong direction, by both groups which were removed automatically before further analysis. The work suggests that recording the gaze using an inexpensive device can have the potential for assisting the neurologists to assess their patients.

## Data Availability

The raw data supporting the conclusions of this article will be made available by the authors, without undue reservation.
